# DSPP dosage affects tooth development and dentin mineralization

**DOI:** 10.1371/journal.pone.0250429

**Published:** 2021-05-26

**Authors:** Dandrich Lim, Ko-Chien Wu, Arthur Lee, Thomas L. Saunders, Helena H. Ritchie

**Affiliations:** 1 Department of Cariology, Restorative Sciences and Endodontics, University of Michigan School of Dentistry, Ann Arbor, Michigan, United States of America; 2 Division of Medical Medicine and Genetics, Department of Internal Medicine, Transgenic Animal Model Core, University of Michigan Medical School, Ann Arbor, Michigan, United States of America; IGBMC/ICS, FRANCE

## Abstract

Dentin Sialoprotein (DSP) and phosphophoryn (PP) are two most dominant non-collagenous proteins in dentin, which are the cleavage products of the DSPP (dentin sialophosphoprotein) precursor protein. The absence of the DSPP gene in DSPP knock-out (KO) mice results in characteristics that are consistent with dentinogenesis imperfecta type III in humans. Symptoms include thin dentin, bigger pulp chamber with frequent pulp exposure as well as abnormal epithelial-mesenchymal interactions, and the appearance of chondrocyte-like cells in dental pulp. To better understand how DSPP influences tooth development and dentin formation, we used a bacterial artificial chromosome transgene construct (BAC-DSPP) that contained the complete DSPP gene and promoter to generate BAC-DSPP transgenic mice directly in a mouse DSPP KO background. Two BAC-DSPP transgenic mouse strains were generated and characterized. *DSPP* mRNA expression in BAC-DSPP Strain A incisors was similar to that from wild-type (wt) mice. *DSPP* mRNA expression in BAC-DSPP Strain B animals was only 10% that of wt mice. PP protein content in Strain A incisors was 25% of that found in wt mice, which was sufficient to completely rescue the DSPP KO defect in mineral density, since microCT dentin mineral density analysis in 21-day postnatal animal molars showed essentially identical mineral density in both strain A and wt mice. Strain B mouse incisors, with 5% PP expression, only partially rescued the DSPP KO defect in mineral density, as microCT scans of 21-day postnatal animal molars indicated a reduced dentin mineral density compared to wt mice, though the mineral density was still increased over that of DSPP KO. Furthermore, our findings showed that DSPP dosage in Strain A was sufficient to rescue the DSPP KO defect in terms of epithelial-mesenchymal interactions, odontoblast lineage maintenance, along with normal dentin thickness and normal mineral density while DSPP gene dosage in Strain B only partially rescued the aforementioned DSPP KO defect.

## Introduction

PP and DSP are the two most dominant non-collagenous proteins in dentin. PP is an extremely acidic protein, and in vitro data suggest PP is a mineral nucleator for dentin mineralization [1]. Following the cDNA cloning of DSP and PP proteins, it became clear that DSP and PP proteins were derived from a single dentin sialophosphoprotein (DSPP) transcript. Actually, DSPP precursor protein is translated from *DSPP* mRNA and secreted into the matrix where it undergoes almost immediate cleavage by a zinc-dependent matrix protease [2] into DSP and PP. Mutations in the human DSPP gene are linked to dentinogenesis imperfecta types II and III [3, 4]. For example, missense mutations likely affected the cleavage of DSPP signal peptide and thus damaged the generation of DSP and PP. A nonsense mutation could generate a short DSP protein without PP expression. Thus, these mutations probably resulted in an obliterated pulp chamber and discoloration. DSPP KO mice showed thin dentin, enlarged pulp chambers, widened predentin, and pulp exposure. These characteristics are consistent with dentinogenesis imperfecta type III [5]. Furthermore, abnormal epithelial-mesenchymal interactions were reported in 1-day and 6-day postnatal DSPP KO mice. Chondrocyte-like cells expressing acidic proteoglycan and collagen type II were identified in DSPP KO mice [6]. These data suggest that DSPP plays a critical role in tooth development, odontoblast lineage, and dentin mineralization.

To better understand how DSPP influences tooth development and dentin formation, we investigated whether the restoration of the DSPP gene in DSPP KO mice could reverse the defects (i.e., thin dentin, enlarged pulp chambers, widened predentin, pulp exposure, epithelial-mesenchymal interactions, and odontoblast lineage). We used a BAC vector that contained the complete DSPP gene and adjacent regulatory regions (BAC-DSPP). The BAC-DSPP transgene would achieve physiologically accurate temporal and spatial expression patterns [7]. Two strains of mice expressing DSPP from a BAC transgene in a DSPP KO background were produced: Strain A and Strain B. Strain A mice expressed *DSPP* mRNA at a level similar to that in wt mice, exhibiting 25% of the wt PP expression and completely rescuing the DSPP KO defect. *DSPP* mRNA levels in Strain B animals were only 10% that of wt mice and PP expression was reduced to 5% of wt mouse PP expression. Strain B mice only partially rescued the DSPP KO defect.

## Results

### Generation of two BAC-wt DSPP transgene mouse strains

**Fig 1:**
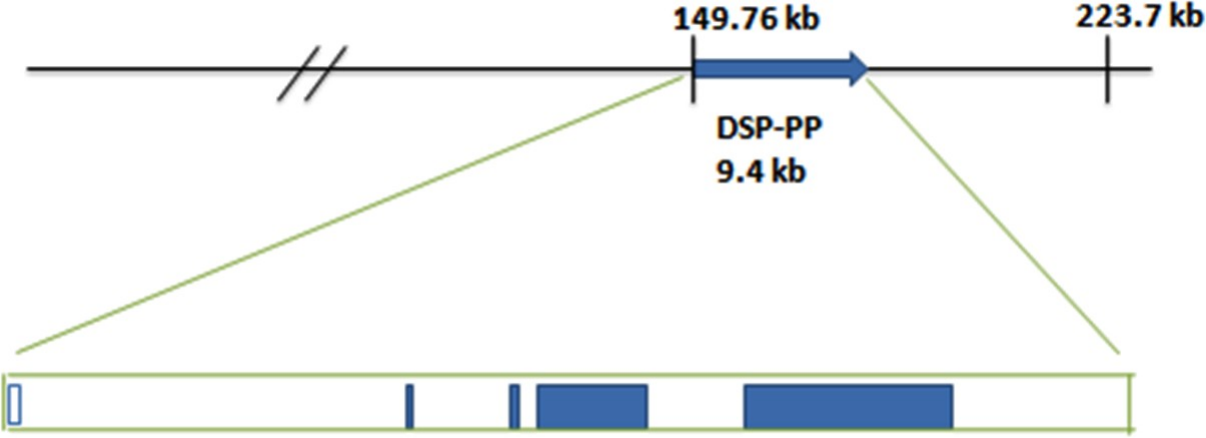
Diagram of mouse bacterial artificial chromosome (BAC) RP23-131C10. This 223,760 bp BAC includes the DSP-PP gene (9.4 kb) with 149.8 kb of upstream and 64.5 kb of downstream sequences. The BAC includes DSPP gene with a complete DSPP promoter.

After microinjections of mouse BAC RP23-131C10 containing wt DSPP gene into C57BL/6J fertilized eggs, the fertilized eggs were implanted in pseudo pregnant mice as described [8]. We identified pups containing the BAC-transgene (BAC^tg/-^) by PCR with BAC specific primers on DNA extracted from tail tip biopsies. We then backcrossed these BAC-transgene mice to DSPP^ko/ko^ mice to produce transgenic mice that carried BAC-wt DSPP in a DSPP^ko/ko^ background (Fig 1).

### *DSPP* mRNA expression from Strain A and Strain B mice

**Fig 2:**
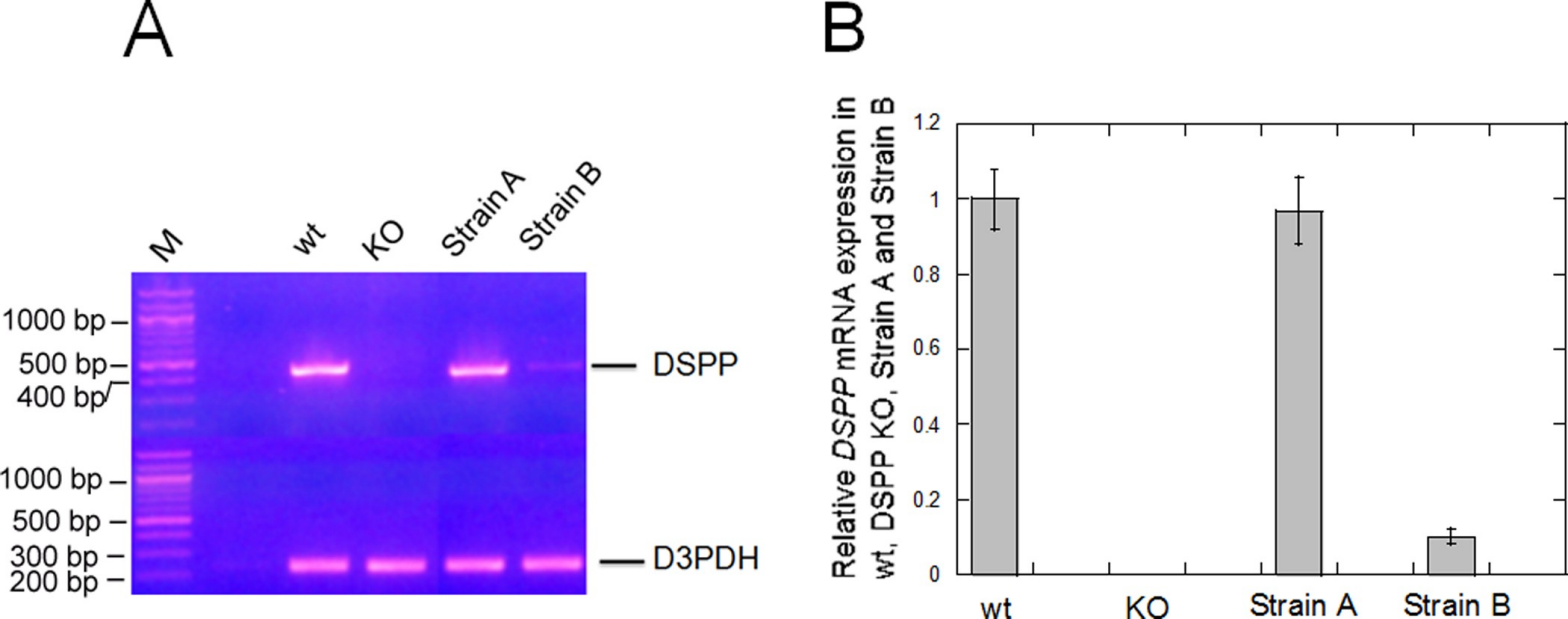
Examination of *DSPP* mRNA expression in wt, DSPP KO, BAC-DSPP transgene Strain A and Strain B teeth. (A) A Thermo Scientific Verso cDNA kit was used to generate cDNA pools from wt, DSPP ko, Strains A and B incisor total RNAs. These cDNA pools were used to perform 30 cycles of polymerase Chain Reactions. Using a pair of PCR primers for DSPP cDNA, a 441 bp DSPP DNA band was detected in wt teeth but was not detected in DSPP KO teeth. A 233 bp glyceraldehyde-3-phosphate dehydrogenase (GAPDH) band was detected in both wt and DSPP KO teeth. (B) Using NIH image J, we determined the relative expression of normalized *DSPP* mRNA and present a graph of the relative expression of *DSPP* mRNA.

BAC-wt DSPP transgene contains the complete DSPP genomic sequence and adjacent regulatory elements. We expected this BAC transgene to drive wt *DSPP* mRNA expression according to its normal temporal and spatial patterns (Fig 1). Indeed, using RT-PCR, Strain A mice showed similar *DSPP* mRNA expression levels to that of wt mouse incisors (Fig 2). Strain B animals showed 10% *DSPP* mRNA expression compared to Strain A and wt mice. No *DSPP* mRNA was detected in DSPP KO mice.

### Levels of PP protein expression

**Fig 3:**
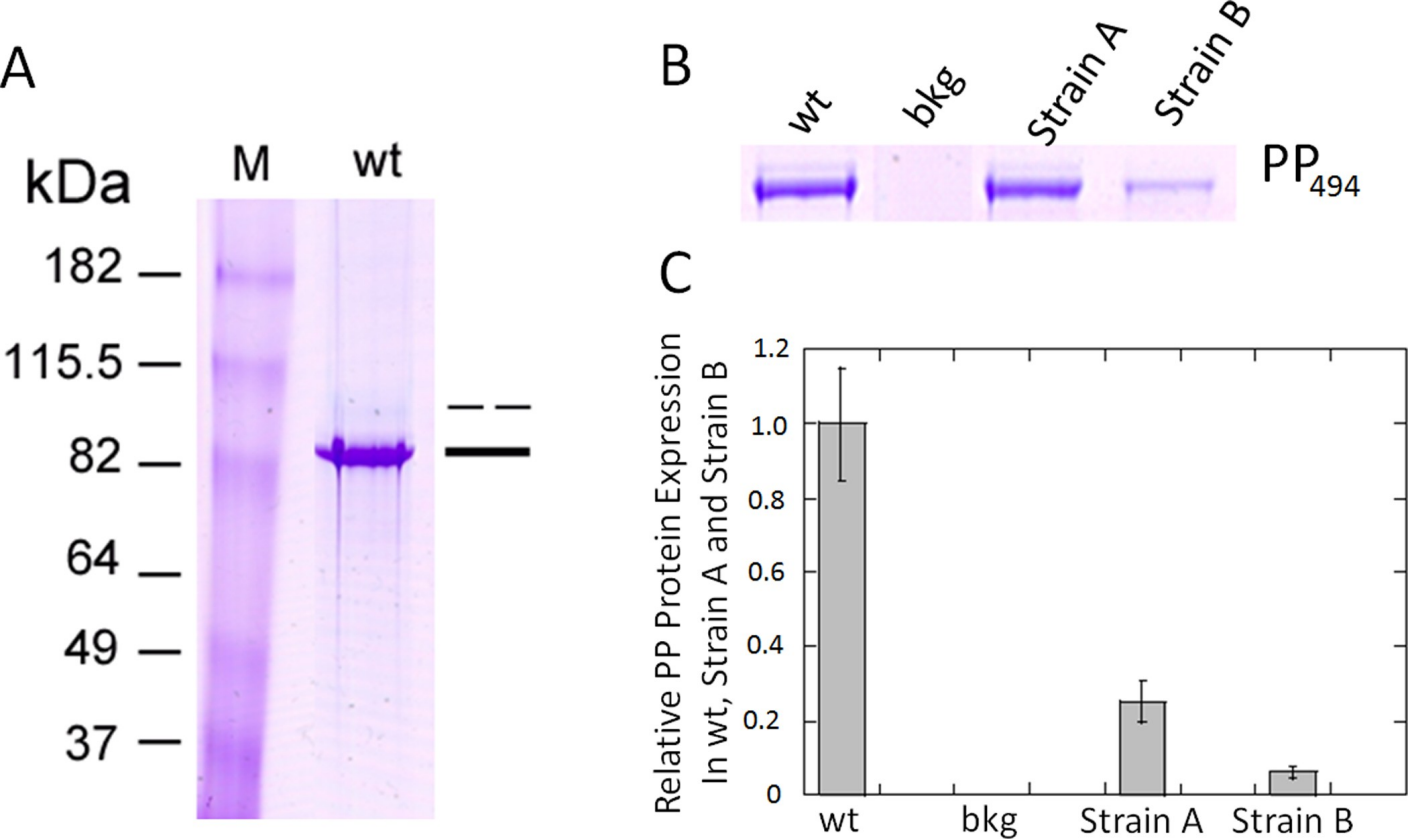
Isolation of DSP and PP from incisor extraction of wt, BAC-DSPP transgene Strain A and Strain B teeth. (A) Lane 1, wt incisor extraction stained with Stains-All showed two bands. The lower major band stained deep blue around 82 kDa was labeled as PP (single bad). The upper minor band stained light blue was labeled as DSP (broken bar). Antibodies against DSP and PP were used to verify the identity of the incisor extract (see S1, S2 and S3 Figs). (B) Tissues were prepared as described in Methods. The incisor extracts from wt (1:10 dilution), Strain A (1:2.5 dilution), and Strain B (1:2.5 dilution) were run on a PAGE gel and stained with Stains-All. This composite image is derived from S4 Fig. (C) NIH J image bar graph of PP_494_ protein expression (corrected for dilutions) from Fig 3B. Since Strain A PP band derived from a 2.5x dilution of the incisor extract, showed a similar intensity of PP from 10x dilution of wt incisor extract. Thus, we concluded that wt TCA extract contain four times more PP than that of Strain A. The NIH J image then showed that Strain B contains 1/5 amount of PP compared to that of Strain A with both bands at 2.5x dilution. In summary, Strain A contained 25% of the wt PP_494_ protein and Stain B contained 5% of the wt PP_494_ protein.

DSPP precursor protein, translated from *DSPP* mRNA, is secreted into the matrix and rapidly cleaved into DSP and PP proteins. DSP and PP are two major noncollagenous acidic proteins in dentin. As shown in Fig 3A, the extracted acidic proteins (stained by Stains-All) consisted of an upper, lighter band and a lower, darker band. Dot blot showed that anti-DSP antibodies detected DSP in wt incisor extraction but did not react with highly phosphorylated protein (HP). Dot blot showed that anti-PP antibodies reacted with wt incisor extraction, recombinant DSPP protein and HP (S1A and S1B Fig). Western blot with anti-DSP antibodies identified DSP band was the upper, lighter band (S2 Fig). Western blot with anti-PP antibodies revealed the lower, darker band was PP (S3 Fig). As the method we used to extract acidic protein is preferentially in favor of PP extraction, the amount of DSP protein obtained from this method is very low. In the wt extract, a high amount of PP showed deep blue color with Stains-All staining and a small amount of DSP showed light blue color. Furthermore, DSPP precursor protein is secreted into the matrix and rapidly cleaved into DSP and PP, whereas DSP is further cleaved by MMP2 and MMP20 into smaller fragments [9]. We therefore focused on and examined PP protein expression as a proxy for DSPP protein expression. Strain A mice PP protein expression levels were 25% of those found in wild type mice (Fig 3B). Since PP is derived from DSPP precursor protein, 25% PP represents 25% DSPP precursor expression in Strain A. Strain B mice PP expression levels were 5% compared to wild type mice (Fig 3C). DSPP KO mice displayed no detectable PP expression (S5 Fig). The disparity between Strain A and B’s percentage mRNA expression levels in comparison to wt and their translated protein level is likely due to BAC DNA fragmentation upon integration into the genome [7, 10]. Transcription initiated from incomplete DSPP genes will result in mRNA molecules that will be not translated into protein although they are detected at the mRNA level. Examples of BAC transgenic mice in which mRNA was detected but not protein expression are described in Van Keuren et al. [8]. This difference in PP protein expression levels among Strain A, Strain B, and wt mice provided an opportunity to explore DSPP’s dosage effect on tooth development and mineralization.

### Histology of Strain A with 25% DSPP protein dosage expression

**Fig 4:**
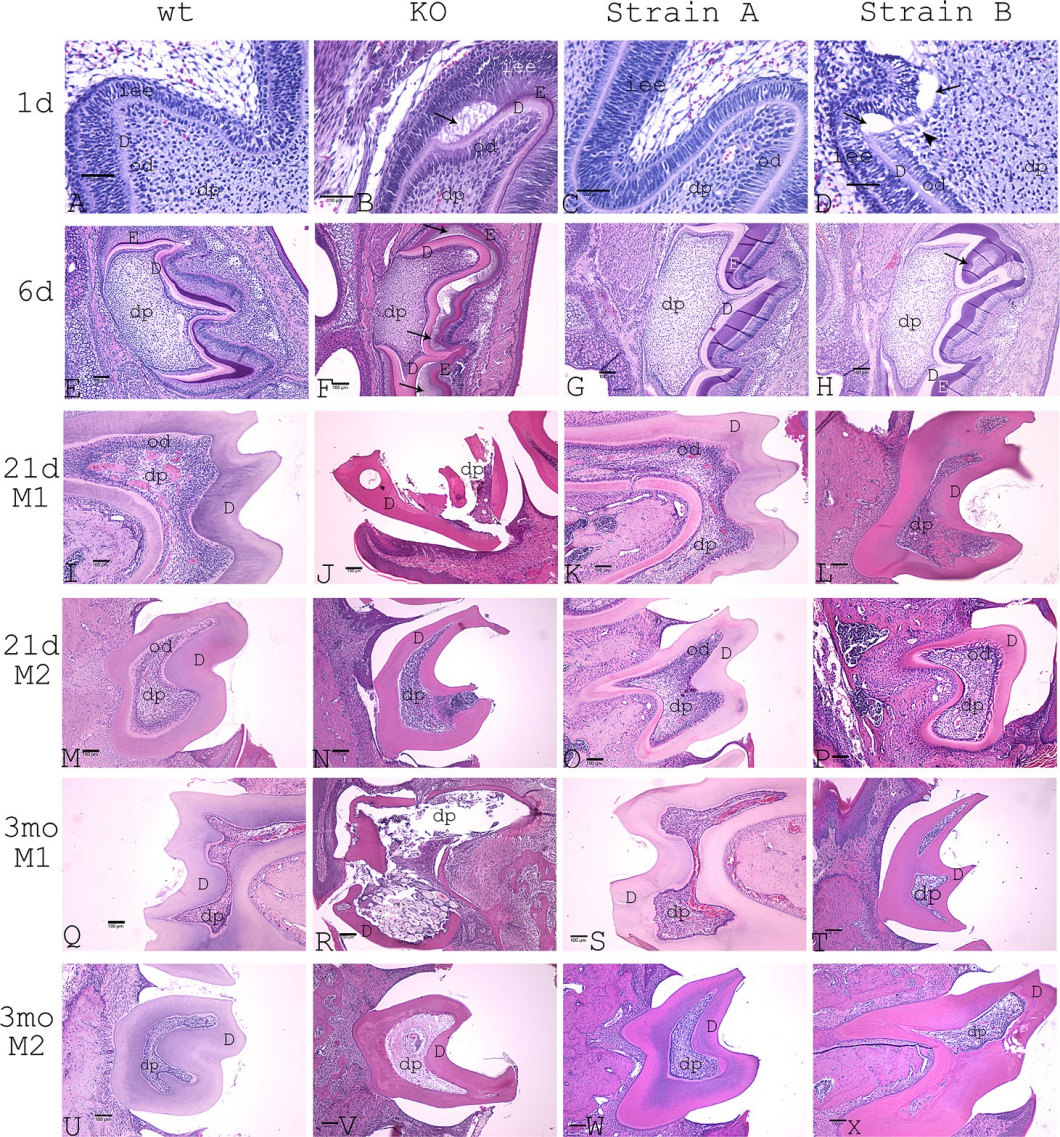
H&E staining of mouse molars at various postnatal time points. A-D: postnatal 1-day molar 1 (M1) at 400x magnification, bar for 50 μm. A: wt. B: DSPP KO. C: Strain A. D: Strain B. Arrows represent the appearance of abnormal dental pulp cells. E-H: postnatal 6-day M1. E: wt. F: DSPP KO. G: Strain A. H: Strain B. dp represents dental pulp. od represents odontoblast layer. D represents dentin. E represents Enamel. iee represents inner enamel epithelium. I-L: postnatal 21-day M1. I: wt. J: DSPP KO mouse. K: Strain A. L: Strain B. M-P: postnatal 21-day M2. M: wt. N: DSPP KO. O: Strain A. P: Strain B. Q-T: postnatal 3-month M1. Q: wt. R: DSPP KO. S: Strain A. T: Strain B. U-X represent postnatal 3-month M2. U: wt. V: DSPP KO. W: Strain A. X: Strain B. E-X: 100x magnification, bar for 100μm.dp represents dental pulp. D represents dentin.

1-day postnatal Strain A mice showed tightly connected epithelial and odontoblast layers. There were no significant differences when compared to wt mice (Fig 4A and 4C). However, DSPP KO mice (i.e., no DSP and no PP protein expression) showed disrupted epithelial and odontoblast layers (Fig 4B).

6-day postnatal wt and Strain A mice displayed tightly connected dentin and enamel layers (Fig 4E and 4G) vs. DSPP KO mice with disrupted epithelial and odontoblast layer (Fig 4F).

21-day postnatal Strain A mice showed intact dentin layers, normal dental pulp chamber sizes, viable dental pulp cells, and a viable odontoblast layer in both molar 1 and molar 2 (M1 and M2) (Fig 4K and 4O). There were no differences between wt(Fig 4I and 4M) and Strain A mice.

3-month postnatal Strain A mice (Fig 4S and 4W) produced no significant differences from wt mice (Fig 4Q and 4U) in terms of odontoblast layer, dental pulp, pulp chamber sizes, dentin thickness, and dentin structure. Thus, 25%PP protein expression (i.e., equivalent to 25% DSPP precursor protein expression) rescued the M1 and M2 of the DSPP KO mice phenotype.

### Histology of Strain B with 5% PP protein

1-day Strain B mice exhibited connected epithelial and odontoblast layers with small interrupting gaps near the cusp region (Fig 4D). DSPP KO mice showed disrupted epithelial and odontoblast layers (Fig 4B).

6-day Strain B mice showed connected dentin and enamel layers but with encircled enamel (indicated by an arrow in Fig 4H).

21-day postnatal Strain B mice displayed intact M1 and M2 dentin layers with no visible dentin fracture (Fig 4L and 4P), while broken tooth was found in DSPP KO mice (Fig 4J). Certain areas in Strain B pulp chambers showed the presence of inflammatory cells (Fig 4L), suggesting the presence of a hidden dentin fracture in adjacent sections. M2 of Strain B showed abnormal but viable odontoblasts and viable dental pulp (Fig 4P). M2 of DSPP KO mice showed damaged tooth tip and severe inflammation (Fig 4N). It is likely the 5% DSPP protein expression contributed to the partial viability of dental pulp and odontoblasts.

3-month postnatal Strain B mice dental pulp cells differentiated into mixed cell populations: odontoblasts and non-odontoblasts (Fig 4T and 4X). Strain B mice still had odontoblasts present in the dental pulp chamber, which indicated some rescue from the 5% DSPP protein because DSPP KO mice had no viable odontoblasts in M1 and M2 (Fig 4R and 4V).

### Chondrocyte-like cells detected in Strain B

**Fig 5:**
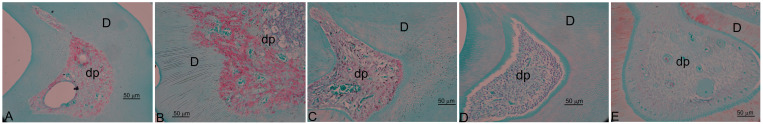
Safranin O and fast green staining to examine acidic proteoglycan expression in DSPP KO, Strain A, Strain B and wt mice at 21-day and 3-month. (A) 21-day DSPP KO M1 showed positive Safranin O staining. (B) 21-day Strain B M1 showed positive Safranin O staining. (C) 3-month Strain B M1 showed positive Safranin O staining. (D) 3-month Strain A showed M1 no Safranin O staining. (E) 3-month wt M1 showed no Safranin O staining. All images at 400x magnification.

**Fig 6:**
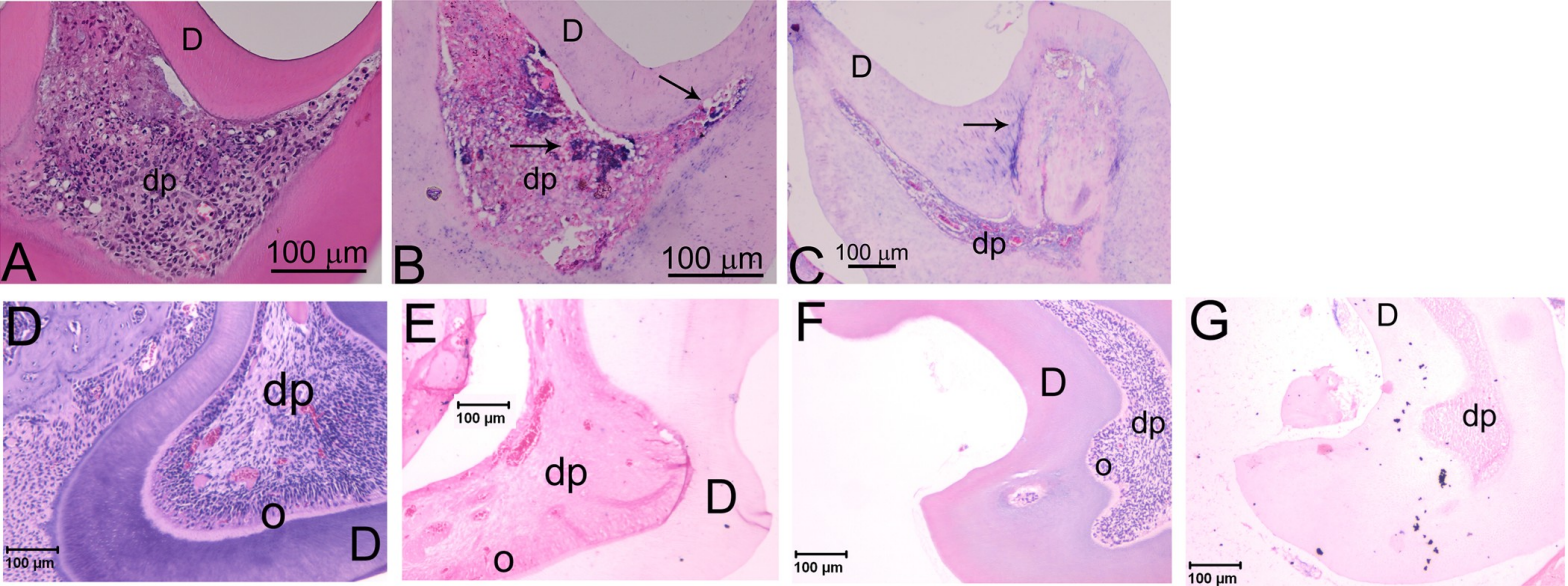
Collagen type II (Col II) expression in Strain B molars. (A) H&E staining of 21-day Strain B M1. 200x magnification. (B) Col II expression in 21-day Strain B M1. 200x magnification. (C) Col II expression in 3-month Strain B M1. 100x magnification. (D) H&E staining of 21-day Strain A M2. 100x magnification. (E) Col II expression of 21-day Strain A M2. 100x magnification. (F) H&E staining of 21-day wt M2. 100x magnification. (G) Col II expression of 21-day wt M2. 100x magnification. dp: dental pulp. D: dentin. Arrows represent Col II expression.

Previously we reported that the non-odontoblast cells in DSPP KO teeth were probably chondrocyte-like cells and confirmed that these non-odontoblast cells expressed acidic proteoglycan and collagen type II, markers for chondrocytes [6]. DSPP is considered to be responsible in maintaining odontoblast lineage [6]. We examined whether the low DSPP expression in Strain B did not maintain the odontoblast lineage, which might result in the appearance of chondrocyte-like cells. Indeed, we observed non-odontoblast cells lining the odontoblast layer and in the dental pulp cells in Strain B teeth. Because Safranin O is used to detect acidic proteoglycan in cartilage, we used Safranin O and Fast Green staining and detected the presence of acidic proteoglycan (as indicated by red staining) in the dental pulp of 21-day DSPP KO M1 (Fig 5A) and Strain B M1 (Fig 5B), as well as 3-monthStrain B M1 (Fig 5C). No Safranin staining was detected in M1 of 3-month Strain A (25% PP) (Fig 5D) and in M1 of 3-month wt (Fig 5E). Thus, acidic proteoglycan was present in the dental pulp of Strain B and DSPP KO M1. No proteoglycan was detected in wt and Strain A M1. To further examine the nature of these abnormal cells in strain B, we used collagen type II antibodies and found these abnormal cells expressed collagen type II as shown in 21-day M1 and in 3-month old M1 (see Fig 6B and 6C). No collagen type II was detected in wt and Strain A dental pulp (Fig 6E and 6G). Therefore, these non-odontoblast cells have the characteristics of chondrocyte-like cells. 5% DSPP protein expression in Strain B did not prevent the appearance of chondrocyte-like cells.

### MicroCT mineral density analysis

**Fig 7:**
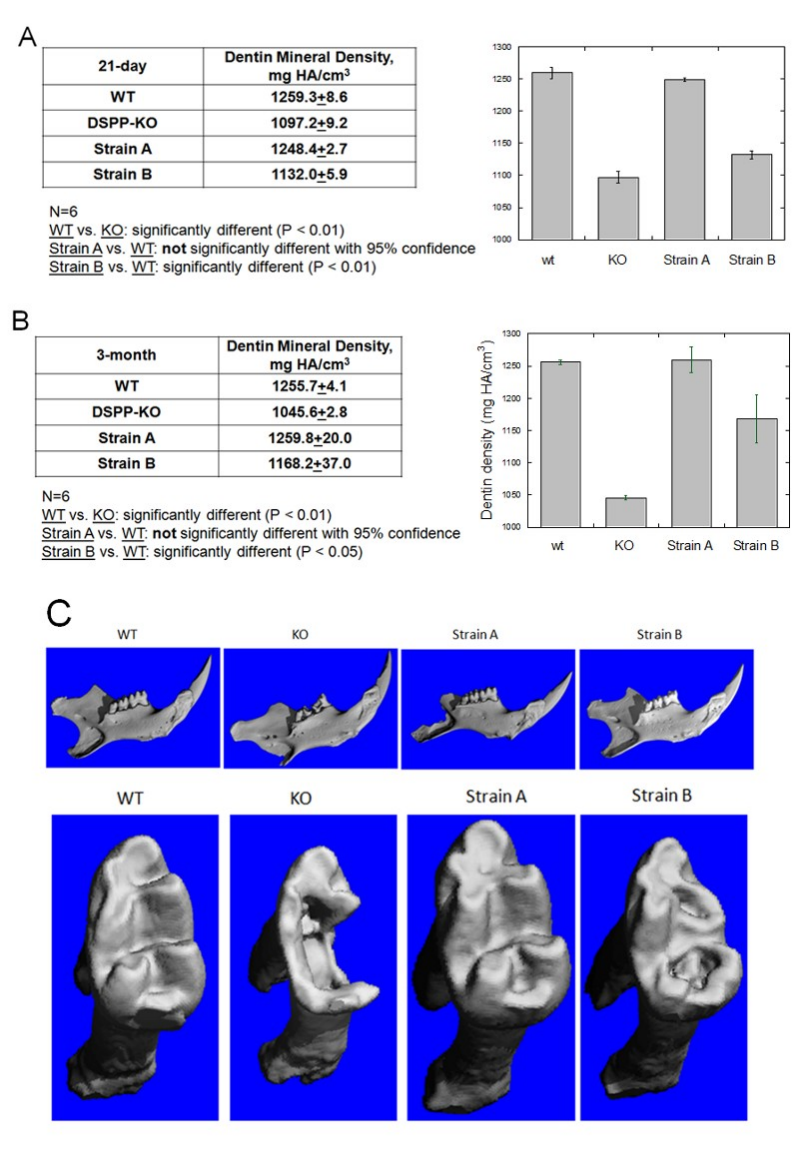
Analysis of mineral density and tooth structure in WT, DSPP KO, BAC-DSPP transgene Strains A and B. (A) Mineral density of M1 dentin at 21-day. (B) Mineral density of M1 dentin at 3-month. (C) 3D reconstruction of 2D microCT images of 21-day old mice. Top panel: incisor and molars. Bottom panel: molar 1. Erosion indicated by arrows.

21-day postnatal Strain A mice, with 25% DSPP protein expression, did not show significant differences in tooth mineral density compared to wt mice (Fig 7A). However, in Strain B mice (with 5% DSPP protein expression), tooth mineral density was significantly lower than Strain A and wt mice. Though when compared to DSPP KO mice, the tooth mineral density of Strain B mice was significantly increased (Fig 7A). In Strain A mice, 25% DSPP protein expression associates with normal dental pulp differentiation into odontoblasts and maintenance of a healthy dental pulp; therefore, viable odontoblasts synthesize sufficient PP to support normal dentin formation. On the other hand, 5%DSPP protein expression partially rescues odontoblast differentiation and mineral density. Similar mineral densities were found in 3-month old Strain A and wt mice. Mineral density of 3-month old Strain B mice was less (Fig 7B). Despite the tooth mineral density of Strain B mice being lower than either Strain A or wt, it was significantly increased (Fig 7B) in postnatal 3-monthmice when compared to DSPP KO mice (Fig 7B).

21-day postnatal Strain A mice molar microCT image shows no significant physical defects compared to wt mice (Fig 7C). This supports our histological findings that 25% DSPP protein expression is sufficient to support long-term tooth development and dentin mineralization. 21-day postnatal Strain B mice molar microCT image shows surface erosion, however it is not as severe as those seen in DSPP KO mice (Fig 7C).

## Discussion

During tooth development, collagen Type I (Col I) is expressed before DSPP [11]. Weinstock and LeBlond [12, 13] showed that phosphoprotein took ~4 hours to reach the mineralization front once secreted from the odontoblasts. In contrast, Col I took ~24 hours to reach the mineralization front. Procollagen I is cleaved outside the cell and forms collagen fibrils, which later undergo self-assembly to form collagen fibers [14].

Zhu et al., used a rat Col Iα1 promoter to drive a normal *DSPP* mRNA expression in their DSPP KO mice. They discovered *DSPP* mRNA expression was 16x higher than that in wt [15]. Col I and DSPP expression patterns are different in terms of time and space [11]. Col I promoter driven DSPP cDNA was not able to correctly express *DSPP* mRNA temporally and spatially in the same pattern as the endogenous DSPP gene. The high level of DSPP expression from the strong Col Iα1 promoter could result in co-expression of DSPP and Col I, which might have unknown effects on collagen maturation as well as the quality of dentin mineralization during tooth development. It would be interesting to compare the collagen assembly and maturation between wt and DSPP KO/ Col Iα1 promoter driven DSPP cDNA transgene mice.

To ensure normal temporal and spatial expression of Col I and DSPP, we created a BAC-DSPP transgene containing a complete DSPP promoter. In Strain Amice, the DSPP promoter in the BAC-DSPP transgene drove *DSPP* mRNA expression at a similar level to endogenous *DSPP* mRNA expression in wt mice. However, StrainB mice only showed 10% of wt mouse *DSPP* mRNA expression. The difference in *DSPP* mRNA expression was likely due to the effect of BAC transgene DNA integration and copy number as had been described in other BAC transgenic mouse models [7, 10, 16]. Future work is needed to determine the exact integration site and genome configuration.

*DSPP* mRNA translates into DSPP precursor protein, which immediately undergoes cleavage via protease BMP1 to generate DSP and PP proteins [2, 17, 18]. Early on, both DSP and PP proteins were considered to play a role in dentin mineralization because DSP and DSPP expression were associated with young odontoblasts and matured odontoblasts during dentin mineralized tissue formation [19–21]. PP is an extremely acidic protein and well established as a mineral nucleator for dentin mineralization [1]. However, the role of DSP has been controversial.

Transgene Strain A expressed 25% PP compared to that of wild type dentin and Strain B expressed 5% PP (see Fig 3). Strain A with 25% PP dosage was likely generated from 25% DSPP precursor protein cleavage. As the precursor protein is cleaved into equal parts DSP and PP, 25% DSP was also likely present in Strain A dentin. Strain B, with 5% PP in dentin, therefore likely contains 5% DSP.

### DSPP protein effect on MicroCT mineral density

As DSP protein was reported as a weak mineralization inhibitor [22], it is unlikely that DSP would enhance mineral formation. PP protein, on the other hand, is considered a major protein responsible for nucleation and dentin mineralization *in vitro* [23–25], likely making its role in dentin mineralization a critical one. 100% PP in wt and 25% PP in Strain A displayed essentially identical dentin mineralization *in vivo*. 25% PP seems to be sufficient to support normal dentin formation. 5% PP in Strain B showed less mineral formation compared to wt; however, it did produce a higher mineral density than that in DSPP KO mice. Thus, a 5% expression still contributed some degree of dentin mineralization.

With respect to physical defects, our microCT images clearly demonstrated that 21-day postnatal Strain A mice molars showed no significant physical defects (Fig 7C), supporting our histological analysis (Fig 4K and 4O). Strain B mice molars displayed surface erosion, but the erosion was less than that seen in DSPP KO mice molars (Fig 7C). And no dentin fractures were observed in Strain B molars (Fig 4L and 4P) as opposed to multiple dentin fractures found in 21-daypostnatal DSPP KO molar teeth (Fig 4J and 4N).

### The effect of DSPP protein dosage on epithelial-mesenchymal interactions and tooth development

*DSPP* mRNA and DSPP protein were reported to be present first in preameloblasts and then in young odontoblasts. DSPP protein was speculated to participate in epithelial-mesenchymal interactions [21]. DSPP KO mice showed abnormal epithelial-mesenchymal interactions (Fig 4B and 4F) [6]. Throughout the 3-month time span, Strain A animal tooth development and dentin mineralization features were indistinguishable from wt animals with respect to epithelial-mesenchymal interactions, odontoblast layer formation, dental pulp and pulp chamber sizes, along with dentin thickness and structure (Fig 4 under wt and Strain A from1d, 6d, 21d, and 3mo). However, we found distinct differences in tooth developmental features between Strain A (25% DSPP protein) and Strain B (5% DSPP protein) animals. For example, Strain B animals began to show small interrupting gaps in epithelial and odontoblast layers near the cusp region as soon as 1-day post-natal examination (Fig 4D). At 21-day, pulp chambers from Strain B animals showed the presence of inflammatory cells, abnormal pulp cells, and fibrocartilage (Fig 4L). Nevertheless, viable odontoblasts and viable dental pulp were present in Strain B animals (Fig 4P), suggesting a DSPP level only 5% of that found in wt animals was sufficient to maintain minimum odontoblast viability.

It is likely DSP and PP proteins generated from DSPP precursor protein are responsible for epithelial-mesenchymal interactions. DSP and PP proteins were reported to promote dental pulp cell differentiation and enhanced *DSPP* mRNA expression [26]. Recombinant DSP protein was reported to promote mesenchymal cell differentiation [27] that resulted in enhanced DSPP and osteocalcin expression. DSP was reported to affect mesenchymal cell differentiation via integrin β6 [28]. While using Col Type I promoter to drive PP expression in DSPP-/- mice, Zhang et al. [29] also reported the transgenic expression of PP partially rescuing the dentin defects and suggested that PP is important for dentin formation and DSP/PP interactions may be important for dentinogenesis. Combined with our data, it is likely 25% DSP and PP proteins in Strain A are sufficient to support normal epithelial-mesenchymal interactions and dental pulp cell differentiation into odontoblasts. However, 5% DSP and PP resulted in defective epithelial-mesenchymal reactions and abnormal cell differentiation.

### Effects of DSPP dosage on odontoblast lineage

We previously identified abnormal chondrocyte-like cells in lacunae in the dental pulp chamber of 1-, 6-, and 21-day postnatal DSPP KO mice [6], which suggested that DSPP is required for normal odontoblast lineage differentiation and that deletion of this protein resulted in the appearance of chondrocyte-like cells in the dental pulp. In this study we looked at DSPP dose effects among Strain A mice (25% DSPP expression of wt animals), Strain B mice (5% DSPP expression of wt animals), and wt animals on odontoblast lineage. We found that 1-day postnatal Strain A mice and wt mice molars both showed normal odontoblast layers with tight connection with inner epithelial layers, and no chondrocyte-like cells were identified. However, molars from Strain B mice did show the appearance of chondrocyte-like cells (indicated by an arrowhead in Fig 4D).

Similarly, molars obtained from Strain A mice at 6-day, 21-day and 3-month postnatal times showed no significant differences with wt mice in terms of viable odontoblast layer and viable dental pulp (Fig 4G, 4K, 4O, 4S and 4W). Yet, in molars from Strain B mice collected at the same stages, dental pulp cells differentiated into mixed populations of cells: odontoblasts and non-odontoblasts (i.e., likely chondrocyte-like cells) (Fig 4H, 4L, 4P, 4T and 4X).

Acidic proteoglycan was detected in M1dental pulp of 21-dayand 3-month Strain B. Furthermore, Col II, a chondrocyte marker was detected in M1 dental pulp of 21-day and 3-month Strain B. We reported that deletion of DSPP protein resulted in the appearance of chondrocyte-like cells in the dental pulp [6]. We speculate that DSPP protein expression is required for maintaining the odontoblast lineage and preventing stem cell differentiation into chondrocyte-like cells. Furthermore, Wu et al. [30] used adenovirus to over-express DSPP in adipose stem cells and showed enhanced expression of genes related to mineralization, such as *Cbfa1*, *Osx*, *BSP*, *OCN* and *DMP1* in these cells, which suggested that DSPP was needed for adipose stem cell differentiation into odontoblast-like cells.

Taken together, DSPP protein can direct stem cells to differentiate into odontoblast lineage cells. From Strain A and Strain B, we conclude that DSPP protein is required for normal odontoblast lineage development. Without DSPP or a low level of DSPP, odontoblast lineage could not be maintained and resulted in the appearance of chondrocyte-like cells.

In summary, two mouse strains with differing amounts of PP (and by inference DSPP) expression were developed using a BAC-DSPP transgene construct: Strain A, which expressed 25% as much DSPP as found in wt mice, and Strain B, which expressed 5% as much DSPP as found in wt mice. Based on histological and microCT bone mineral density data over the course of a 6-month tooth developmental time frame, we found that DSPP expression levels 25% of those in wt animals were sufficient to restore normal tooth development and dentin mineralization in DSPP KO mice, while PP expression levels 5% of those in wt animals were not capable of doing so. These results support our previous study demonstrating that DSPP is required for normal odontoblast lineage differentiation and that deletion of this protein (i.e., in DSPP-KO mice) results in the appearance of chondrocyte-like cells in dental pulp [6]. Taken together, these two studies demonstrate the interdependence of odontoblast lineage differentiation and subsequent tooth development with DSPP protein expression.

## Materials and methods

### Wild type (wt) and dentin sialophosphoprotein (DSPP) knockout (KO) mice

We used C57BL/6J mice as the experimental model. Wt mice were obtained from the Jackson Laboratory (Bar Harbor, ME). DSPP KO mice (B6; 129-Dspptm1Kul/Mmnc) were obtained from Mutant Mouse Resource and Research Center (MMRRC), UNC (Chapel Hill, NC) [5]. Wt and DSPP KO mice were fed LabDiet Formula 5008 (PMI Nutrition International, LLC, Brentwood, MO) to maintain nutrition and reduce tooth wear. All animal colonies were handled and maintained in accordance with the guidelines and protocols approved by the University Committee on Use and Care of Animals (IACUC, Protocol number:10401–1).

### Generation of the BAC-wt DSPP transgene in DSPP KO mice

Mouse BAC RP23-131C10 containing wt DSPP (Fig 1) was obtained from Children’s Hospital Oakland Research Institute, and injected into C57BL/6J fertilized eggs in order to generate BAC transgenic mice (BAC^tg/-^) as described in van Keuren, et al. [8]. DSPP^ko/ko^ mice, purchased from MMRRC [5], were backcrossed to BAC transgenic mice to produce transgenic mice expressing the DSPP wt transcript in a DSPP^ko/ko^ genetic background (BAC^tg/-^, DSPP^ko/ko^). We used BAC specific primers: forward primer 5’-AATGTCAAGCCCCGGCCAGC-3’ and reverse primer 5’-ACGCGATTGCGACGTGCTGA-3’ to determine whether our pups contained the BAC transgene. We used DSPP forward primer 5’-GTATCTTCATGGCTGTTGCTTC-3’ and DSPP reverse primer 5’-TGTGTTTGCCTTCATCGAGA-3’ to detect the DSPP transgene mRNA. We used DSPP forward primer and LacZ reverse primer 5’-CCTCTTCGCTATTACGCCAG-3’ to detect the DSPP^ko/ko^ mutation.

### BAC transgene mice

Two independent BAC transgenic founders were used to establish the strains of BAC-DSPP^ko/ko^ mice: Strain A and Strain B.

### Detection of *DSPP* mRNA expression

21-day wt, DSPP KO, and Strains A and B mice were euthanized with carbon dioxide and their mandibles collected. Teeth were extracted using a dissecting microscope in order to eliminate bone, periodontal ligaments, and muscular tissue. Both mandibular teeth from the same mouse were used for each RNA extraction. The teeth were ground by mechanical force in liquid nitrogen, and RNA was extracted using Trizol (Invitrogen, Life technologies, USA). Thermo Scientific Verso cDNA Kit was used to generate cDNA pools from the RNA samples extracted from incisor teeth of wt, DSPP KO, Strains A and B mice by following the manufacturer’s protocol. Mouse *DSPP* mRNA expression level was determined via 30 cycles of PCR with the reverse-transcription generated cDNAs. using the following DSPP primers: 5’-TGAAGAAGGCGACAGTACCC-3’ (forward) and 5’-TCACTTTCGTCACTTCCGTTAG-3’ (reverse), which produces a 441 bp DNA fragment. Similarly, mouse glyceraldehyde-3-phosphatedehydrogenase (GAPDH) gene expression level was determined with the following primers: 5’-GGTGAAGGTCGGTGTGAACG-3’ (forward) and 5’-CTCGCTCCTGGAAGATGGTG-3’ (reverse), which yielded a 233 bp band. GAPDH served as an internal control.

### Incisor PP protein extraction and PAGE

21-day wt, DSPP KO, and Strains A and B mice were euthanized and both mandibles were collected. Incisors were extracted using a dissecting microscope to eliminate bone, periodontal ligament, and muscular tissue. For each mouse genotype, eight mandibular incisors were collected for protein extraction, and the weight of incisors was recorded. The incisors were washed in 1X PBS with 1X proteinase inhibitors (PIs, Amresco, Solon, OH) and 0.3 mM PMSF, and then ground to powder with mechanical force in liquid nitrogen. The powdered tissues containing both enamel and dentin were transferred to solubilization buffer (150 mM NaCl, 20 mM Tris-HCl pH 7.5, 1% NP-40, 5 mM EDTA) with 1X PIs and 0.3 mM PMSF, and then TCA was added to the mixture to reach a final concentration of 5% for dentin protein extraction. Dentin matrix proteins included collagen type I and non-collagenous proteins such as DSP, PP, osteocalcin, and osteopontin. Under these extraction conditions, acidic proteins were soluble in 5% TCA while nonacidic proteins were precipitated. The extraction was carried out for one hour at room temperature, and the mixture was centrifuged at 12,000g for five minutes. The supernatants were then collected, neutralized and precipitated with calcium by adding 3M Tris-HCl pH 8.8 and 1M CaCl_2_, then centrifuged at 12,000g for five minutes. The supernatant was aspirated and the precipitate was re-suspended in 0.1M EDTA pH 8.0. The extracted dentin matrix proteins were analyzed by polyacrylamide gel electrophoresis with Stains-All staining as described previously [31]. To determine the presence of DSP and PP in the extracted incisor matrix, first dot blot (a simplification of western blot method) was used with anti-DSP and anti-PP antibodies. Later western blot (as described previously [32]) with anti-DSP antibodies and anti-PP antibodies was performed to confirm the identities of DSP and PP in the extracted dentin matrix proteins. Anti-DSP antibodies was a gift from Dr. Chunlin Qin, Baylor College of Dentistry at Dallas, Tx. Anti-PP antibodies was generated by Dr. Ritchie’s lab.

### Micro-computed tomography (microCT) analysis of dentin mineral density

Wt, DSPP KO, and BAC-DSPP Strain A and Strain B mandibles were extracted as described above and fixed in 10% formalin at room temperature for 48 hours. Fixed mandibular samples were scanned at the University of Michigan School of Dentistry microCT Core Facility. Specimens were mounted in 1% agarose and placed in a 19mm diameter tube and scanned over the entire length of the left mandible using a microCT system (QCT100 Scanco Medical, Bassersdorf, Switzerland). Scan settings were: voxel size 16 Qm, 70 kVp, 114 QA, 0.5 mm AL filter, and integration time 500 ms. 3D reconstruction was created from microCT2D images. Analysis was performed using the manufacturer’s evaluation software. As the grayscale is a histogram of the density, a fixed global threshold of 30% (300 on a grayscale of 0–1000) was used to segment bone from nonbone; upper threshold of 60% was used to segment dentin from enamel. Contours were manually drawn around the first molar of each specimen (approximately 100 image slices per molar).

### Statistical analysis

Results are presented as means ± standard error of the mean (S.E.M.). An analysis of variance (ANOVA) on the groups was performed to confirm that there is a significant difference amongst them overall. Then pairwise comparisons, adjusted by Tukey’s test, were made between all groups by using the program Prism (GraphPad Software, San Diego, CA) in the Center for Statistical Consultation and Research Center of The University of Michigan. Data were plotted using KaleidaGraph (Synergy Software, Reading, PA).

### Tissue preparation and histological analyses

Tissues were collected from wt, BAC-DSPP Strains A and B, and DSPP KO mice at various ages. For 1-day and 6-day-old mice, the entire head was collected; for 21-day and 3-month-old mice, only mandibles were collected. Tissues were fixed with 10% formalin at room temperature for 48 hours, then demineralized with 0.25M ethylene diamine tetraacetic acid (EDTA, pH 7.4) at room temperature for 15 days. Then, tissues were dehydrated, paraffin-embedded, sectioned at 5 μm thickness, stained with Hematoxylin & Eosin (Sigma-Aldrich, St. Louis, MO.; H&E), and mounted with Permount (Fisher Scientific, Waltham, MA). Images were taken using a Nikon Eclipse E400 microscope (Tokyo, Japan) and SPOT RT Slider Microscope Camera (Diagnostic Instruments, Sterling Heights, MI). Scale bars for 200X, 400X, and 1,000X magnifications were added to the images using the manufacturer’s software.

## Supporting information

S1 Fig Dot blot hybridization with anti-DSP and anti-PP antibodies. (A) Dot blot hybridized with anti-DSP antibodies. Wt (2 ml): Isolated dentin protein from wt incisors. HP (0.5 mg): highly phosphorylated Rat PP. DSP was detected in wt. No DSP was detected in HP. (B) Dot blot with anti-PP antibodies. Wt (2 ml). wt at 1:5 dilution. Recombinant DSP-PP. HP (0.5 mg). PP was detected in wt, recombinant DSP-PP and HP.
(PDF)

S2 Fig Western blot analyses of the minor band isolated from wt incisors as DSP protein. Using anti-DSP antibodies, the minor band was DSP protein.
(PDF)

S3 Fig Western blot analyses of the major band isolated from wt incisors as PP protein. Using anti-PP antibodies, the lower major blue band was PP protein. Above 115.5 kDa, the detected bands likely represent PP dimers and trimers.
(PDF)

S4 Fig Isolation and Stains-All staining of acidic proteins from mouse incisor extraction of wt, BAC-DSPP transgene Strain A and Strain B mice. The acidic protein extracted from mouse incisors from BAC-DSPP transgene Strain A, Strain B and wt was shown with Stains-All staining. Lanes 1–4: samples of Strain A were loaded at 1:2.5x, 1:5x, 1:10x and 1:20x dilutions. Lanes 5–6: samples of Strain B were loaded at 1:2.5x and 1:10x dilutions. Lanes 7–8: samples of wt was loaded at 1:5x and 1:10x dilutions. A major blue band located above 82 kDa was present in all samples. A second light blue band with a higher molecular weight compared to that of the major blue band was detected in wt sample. Since TCA extraction method favored PP isolation, a major PP band was displayed in all sample. Likely the second light blue band was DSP protein.
(PDF)

S5 Fig Isolation and Stains-All staining of acidic proteins from mouse incisor extraction of wt and DSPP KO mice. M: size marker. Lane 1: wt sample was loaded without dilution. A major blue band was detected around 82 kDa, which represented PP. Lane 2: DSPP KO sample was loaded without dilution. No blue PP band was detected.
(PDF)
