# Supplementary material for: DSPP dosage affects tooth development and dentin mineralization
Source: PLoS One. 2021 May 26;16(5):e0250429. doi: 10.1371/journal.pone.0250429 (PMC8153449; doi:10.1371/journal.pone.0250429)
Supplement: S1 Fig — (A) Dot blot hybridized with anti-DSP antibodies. Wt (2 ml): Isolated dentin protein from wt incisors. HP (0.5 mg): highly phosphorylated Rat PP. DSP was detected in wt. No DSP was detected in HP. (B) Dot blot with anti-PP antibodies. Wt (2 ml). wt at 1:5 dilution. Recombinant DSP-PP. HP (0.5 mg). PP was detected in wt, recombinant DSP-PP and HP. (PDF) [file pone.0250429.s001.pdf]

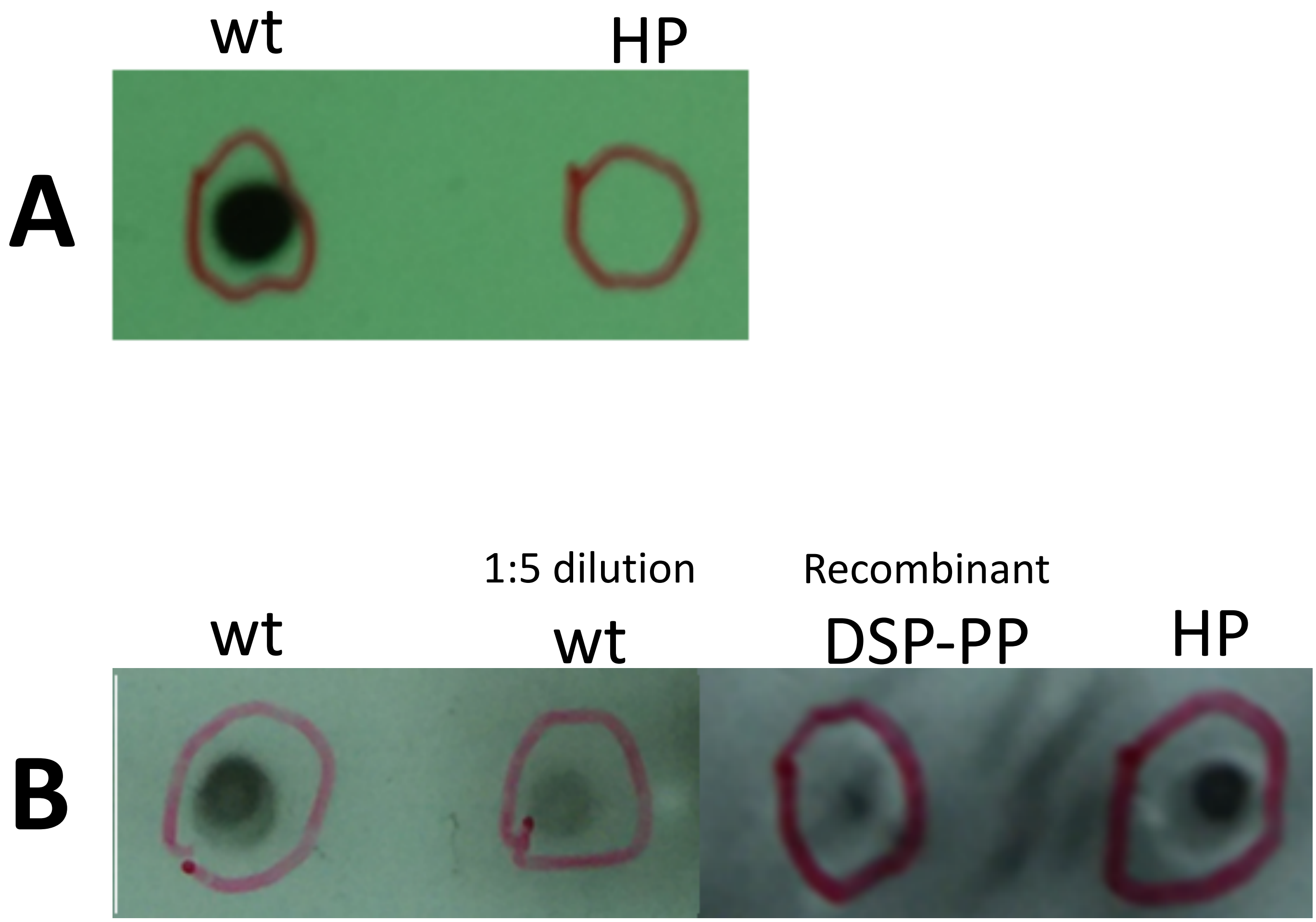

S1 Fig. Dot blot hybridization with anti-DSP and anti-PP antibodies.

(A) Dot blot hybridized with anti-DSP antibodies. Wt (2 ml): Isolated dentin protein from wt incisors. HP (0.5 mg): highly phosphorylated Rat PP. DSP was detected in wt. No DSP was detected in HP. (B) Dot blot with anti-PP antibodies. Wt (2 ml). wt at 1:5 dilution. Recombinant DSP-PP. HP (0.5 mg). PP was detected in wt, recombinant DSP-PP and HP.
