# Supplementary material for: DSPP dosage affects tooth development and dentin mineralization
Source: PLoS One. 2021 May 26;16(5):e0250429. doi: 10.1371/journal.pone.0250429 (PMC8153449; doi:10.1371/journal.pone.0250429)
Supplement: S2 Fig — Using anti-DSP antibodies, the minor band was DSP protein. (PDF) [file pone.0250429.s002.pdf]

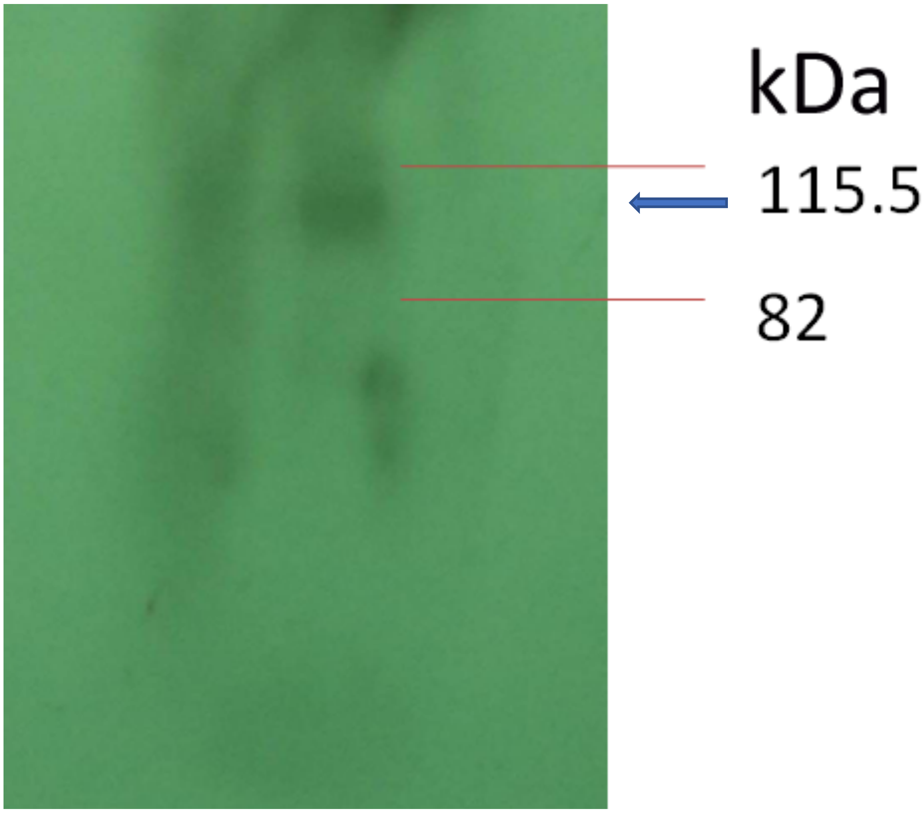

S2 Fig. Western blot analyses of the minor band isolated from wt incisors as DSP protein. Using anti-DSP antibodies, the minor band was DSP protein.
