# Supplementary material for: DSPP dosage affects tooth development and dentin mineralization
Source: PLoS One. 2021 May 26;16(5):e0250429. doi: 10.1371/journal.pone.0250429 (PMC8153449; doi:10.1371/journal.pone.0250429)
Supplement: S3 Fig — Using anti-PP antibodies, the lower major blue band was PP protein. Above 115.5 kDa, the detected bands likely represent PP dimers and trimers. (PDF) [file pone.0250429.s003.pdf]

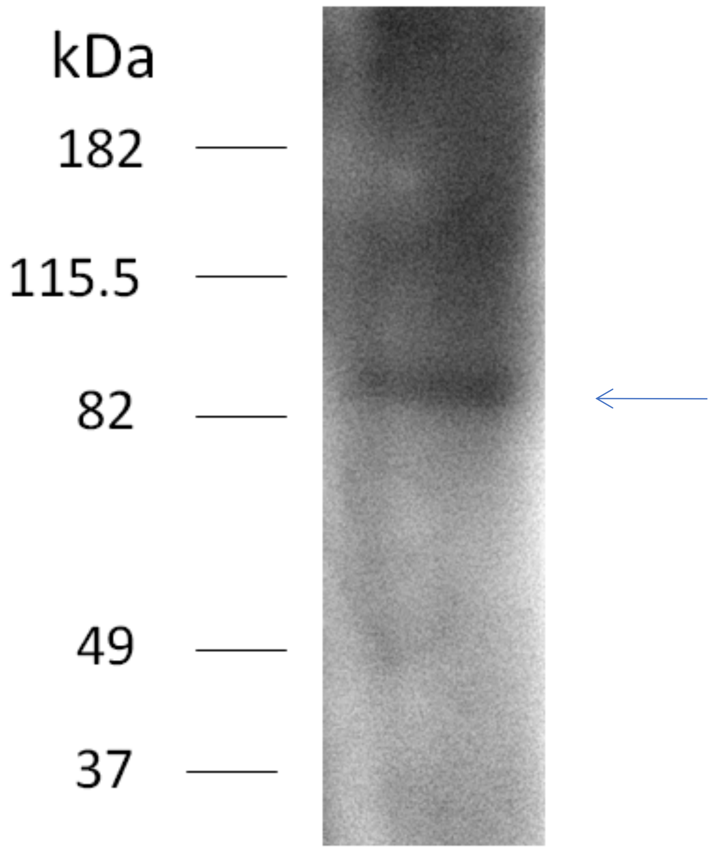

S3 Fig. Western blot analyses of the major band isolated from wt incisors as PP protein. Using anti-PP antibodies, the lower major blue band was PP protein. Above 115.5 kDa, the detected bands likely represent PP dimers and trimers.
