# Supplementary material for: DSPP dosage affects tooth development and dentin mineralization
Source: PLoS One. 2021 May 26;16(5):e0250429. doi: 10.1371/journal.pone.0250429 (PMC8153449; doi:10.1371/journal.pone.0250429)
Supplement: S4 Fig — The acidic protein extracted from mouse incisors from BAC-DSPP transgene Strain A, Strain B and wt was shown with Stains-All staining. Lanes 1–4: samples of Strain A were loaded at 1:2.5x, 1:5x, 1:10x and 1:20x dilutions. Lanes 5–6: samples of Strain B were loaded at 1:2.5x and 1:10x dilutions. Lanes 7–8: samples of wt was loaded at 1:5x and 1:10x dilutions. A major blue band located above 82 kDa was present in all samples. A second light blue band with a higher molecular weight compared to that of the major blue band was detected in wt sample. Since TCA extraction method favored PP isolation, a major PP band was displayed in all sample. Likely the second light blue band was DSP protein. (PDF) [file pone.0250429.s004.pdf]

Fig 3B Lane WT, Strain A, Strain B

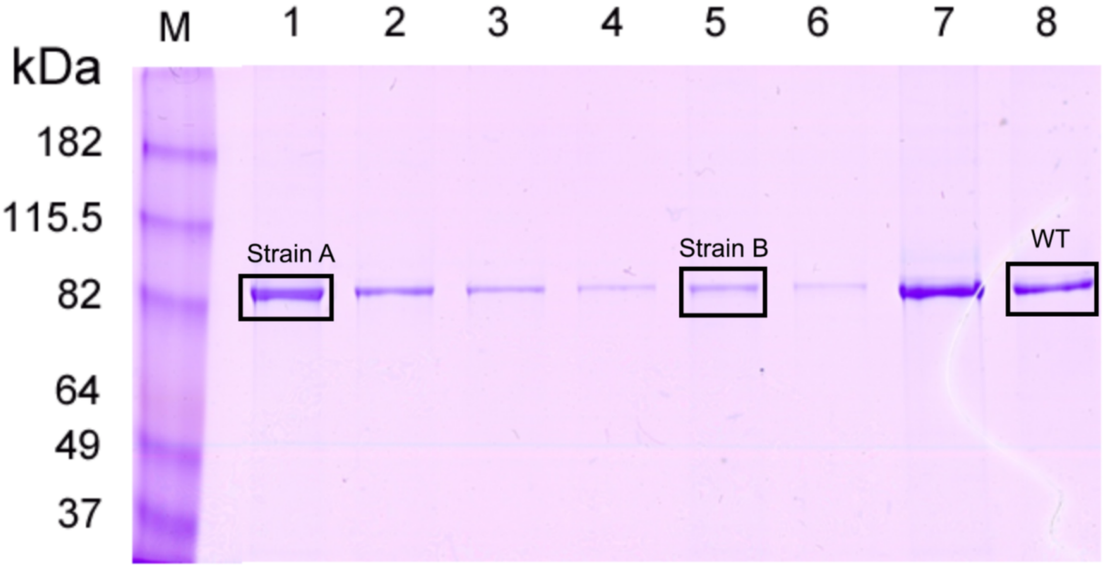

S4 Fig. Isolation and Stains-All staining of acidic proteins from mouse incisor extraction of wt, BAC-DSPP transgene Strain A and Strain B mice. The acidic protein extracted from mouse incisors from BAC-DSPP transgene Strain A, Strain B and wt was shown with Stains-All staining. Lanes 1-4: samples of Strain A were loaded at 1:2.5x, 1:5x, 1:10x and 1:20x dilutions. Lanes 5-6: samples of Strain B were loaded at 1:2.5x and 1:10x dilutions. Lanes 7-8: samples of wt was loaded at 1:5x and 1:10x dilutions. A major blue band located above 82 kDa was present in all samples. A second light blue band with a higher molecular weight compared to that of the major blue band was detected in wt sample. Since TCA extraction method favored PP isolation, a major PP band was displayed in all sample. Likely the second light blue band was DSP protein.
