# Supplementary material for: DSPP dosage affects tooth development and dentin mineralization
Source: PLoS One. 2021 May 26;16(5):e0250429. doi: 10.1371/journal.pone.0250429 (PMC8153449; doi:10.1371/journal.pone.0250429)
Supplement: S5 Fig — M: size marker. Lane 1: wt sample was loaded without dilution. A major blue band was detected around 82 kDa, which represented PP. Lane 2: DSPP KO sample was loaded without dilution. No blue PP band was detected. (PDF) [file pone.0250429.s005.pdf]

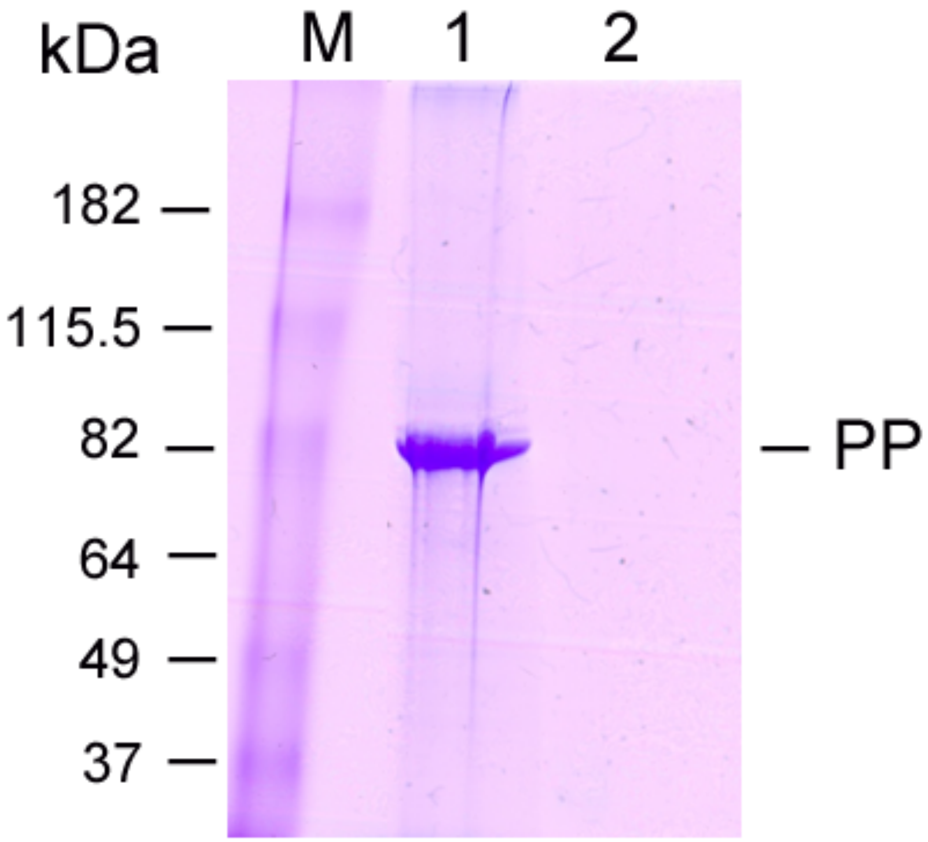

S5 Fig. Isolation and Stains-All staining of acidic proteins from mouse incisor extraction of wt and DSPP KO mice. M: size marker. Lane 1: wt sample was loaded without dilution. A major blue band was detected around 82 kDa, which represented PP. Lane 2: DSPP KO sample was loaded without dilution. No blue PP band was detected.
